# Trophic provisioning and parental trade-offs lead to successful reproductive performance in corals after a bleaching event

**DOI:** 10.1038/s41598-022-21998-4

**Published:** 2022-11-04

**Authors:** Lisa J. Rodrigues, Jacqueline L. Padilla-Gamiño

**Affiliations:** 1 Department of Geography and the Environment, Villanova University, 800 Lancaster Avenue, Villanova, PA 19085 USA; 2 School of Aquatic and Fishery Sciences, University of Washington, 1122 NE Boat Street, Seattle, WA 98105 USA

**Keywords:** Physiology, Marine biology

## Abstract

Warming ocean temperatures are severely compromising the health and resilience of coral reefs worldwide. Coral bleaching can affect coral physiology and the energy available for corals to reproduce. Mechanisms associated with reproductive allocation in corals are poorly understood, especially after a bleaching event occurs. Using isotopic labeling techniques, we traced the acquisition and allocation of carbon from adults to gametes by autotrophy and heterotrophy in previously bleached and non-bleached *Montipora capitata* and *Porites compressa* corals. Experiments revealed that both species: (1) relied only on autotrophy to allocate carbon to gametes, while heterotrophy was less relied upon as a carbon source; (2) experienced a trade-off with less carbon available for adult tissues when provisioning gametes, especially when previously bleached; and (3) used different strategies for allocating carbon to gametes. Over time, *M. capitata* allocated 10% more carbon to gametes despite bleaching by limiting the allocation of carbon to adult tissues, with 50–80% less carbon allocated to bleached compared to non-bleached colonies. Over the same time period, *P. compressa* maintained carbon allocation to adult tissues, before allocating carbon to gametes. Our study highlights the importance of autotrophy for carbon allocation from adult corals to gametes, and species-specific differences in carbon allocation depending on bleaching susceptibility.

## Introduction

Understanding variation in resource allocation is important for identifying traits that enhance organismal survival during stressful environmental conditions^1^ and for predicting population responses to global climate change^2^. How parents manage exposure to environmental stressors can have a significant impact on the ability of their offspring to cope with similar stressors in the future^3–7^. Since reef-building corals live close to their thermal tolerance limits, they are especially sensitive to anthropogenic stressors, including ocean warming due to climate change^8^. Coral bleaching or the loss of photosynthetic symbionts due to thermal stress is expected to become more frequent and severe worldwide^9,10^, with annual bleaching predicted for most reefs by 2050^11^. More than 80% of reefs are expected to experience harmfully frequent bleaching events through the end of this century^12,13^.This increase in the frequency and severity of bleaching is predicted to affect coral physiology, compromising both successful reproductive cycles and the potential for species adaptation. To enhance our predictive capacity about the future of coral reef ecosystems, it is critical to assess recovery strategies and the reproductive potential of corals following environmental stress (reviewed in^6^).

In corals, delayed gametogenesis, decreased egg size, low fecundity, and reduced likelihood of spawning may occur when elevated sea surface temperatures cause corals to bleach^14–22^. However, some species can continue sexual reproduction despite bleaching^15,18,19,23,24^. Successful reproduction after bleaching may be due to the mixotrophic ability of adult corals to obtain carbon from different sources. Both autotrophic and heterotrophic pathways are relied upon by adult corals as they recover from bleaching events^25^. In adult colonies it is well known that coral bleaching results in decreased rates of photosynthesis and increased metabolism of stored lipid reserves^26,27^ that in some species may be supplemented by increased rates of heterotrophy^25,28,29^. While trophic strategies vary among corals and are thought to influence bleaching resistance and thermal tolerance in adults^30^, their influence on gamete development and any resulting parental trade-offs remains largely uninvestigated. Carbon acquisition by parents and the provisioning of carbon to their offspring may contribute to the varied outcome in coral reproduction following bleaching.

Carbon stored as lipids in eggs^31^ is particularly important for species with lecithotrophic development, including corals, as larval nutrition is limited to resources contained within the egg^32^. To date, it is not known whether the carbon that is translocated to coral eggs is the result of autotrophic or heterotrophic carbon acquisition by the parent. Baumann et al.^33^ hypothesized that heterotrophic carbon may be disproportionately allocated to lipids in released coral eggs, independent of bleaching status of the parent. In the coral *Pocillopora verrucosa*, heterotrophy seemed to negatively impact gamete development^34^, while there was a positive relationship in the gorgonian *Paramuricea clavata*^35^. To date, only one prior study has traced photosynthate products from adult coral colonies of *Stylophora pistillata* to their released planulae (i.e., larvae)^36^. In this brooding species with fertilization and larval development occurring within parental polyps, there was greater transfer of autotrophic carbon from parents to planula than to eggs^36^, suggesting that the species may prioritize transfer of fixed carbon to offspring during embryogenesis. It is unknown how carbon is provisioned by corals that are broadcast spawners, where fertilization occurs in the water column after release of eggs/sperm from polyps. Furthermore, it is unknown how carbon is provisioned by parent to offspring following environmental stress for any coral reproductive strategy.

**Figure 1:**
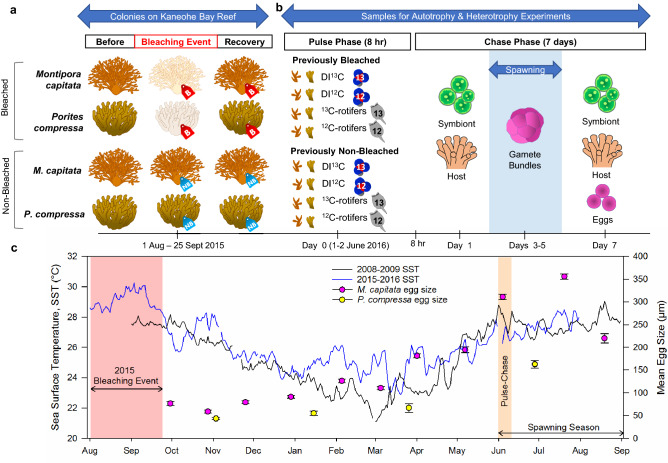
Experimental design and reproductive cycle in Hawaiian corals. (**a**) Bleached and non-bleached colonies were tagged during a bleaching event in Kāne'ohe Bay in Aug-Sept 2015^41^ and monitored for 8 months until recovery. (**b**) In June 2016, four fragments were collected from each colony and the pulse phase of the experiment was conducted for 8 h to evaluate carbon acquisition by autotrophy (DI^13^C or DI^12^C) or heterotrophy (^13^C-rotifers or ^12^C-rotifers). Allocation of ^13^C was measured during 7 days of a chase phase in symbiont cells, host tissue, and eggs. (**c**) Mean daily sea surface temperature (SST) from NOAA Station MOKH1 (21° 25′ 59" N 157° 47′ 23" W) for 2008–2009, a non-bleaching year when egg size for *Monitpora capitata* was previously reported^42^, and 2015–2016, when bleaching occurred. SST is compared to egg size (mean ± standard error) for *M. capitata*^42^ and *Porites compressa* (Padilla-Gamiño, unpublished data).

To better understand the link between the type of carbon acquisition by parents (autotrophic vs. heterotrophic) and subsequent allocation to offspring, we performed a series of experiments to follow carbon from parents to eggs in two coral species. We compared provisioning of ^13^C in colonies that had previously bleached and recovered with those colonies that did not bleach during thermal stress (Fig. 1). Specifically, we: (1) traced carbon allocation in adult corals after a bleaching event; (2) traced parental provisioning of carbon in adult corals to gametes; (3) assessed provisioning to gametes via autotrophic and heterotrophic pathways; and (4) focused on broadcast spawners with hermaphroditic (*Montipora capitata*) and gonochoric (*Porites compressa*) reproductive strategies.

## Methods

### Coral species

We examined carbon acquisition and allocation in two Hawaiian reef-building corals, *Montipora capitata* and *Porites compressa*. Both coral species are dominant in Kāne'ohe Bay (O'ahu, Hawai'i, USA), where the study was performed. *Montipora capitata* is a simultaneous hermaphrodite with buoyant gamete (i.e., egg-sperm) bundles and surface water fertilization^37,38^. *Porites compressa* is a gonochoric spawner with neutrally buoyant eggs and fertilization within the water column^39^. To assess parental provisioning to eggs in both species, we focused analyses on female colonies of *P. compressa* in this study. Both of these species spawn during the summer, although *P. compressa* has a more extended (summer through fall) and less synchronized spawning period than *M. capitata*^40^*. Montipora capitata* has higher settlement rates than *P. compressa* but early settlers have higher mortality and slow growth compared to *P. compressa*^40^.

### Experimental design

Our study was designed to follow the timing of both thermal stress (a naturally occurring bleaching event^41^) and the coral reproductive cycle (development of gametes and release during the spawning season^42^). Our study site, reef K4 (21°26′ 36.6″ N, 157° 48′ 21.6″ W), is a fringing reef located in the central part of Kāne'ohe Bay where water has a residence time of 10–20 days^43^. Flow is wave driven and influenced by wind driven long-shore currents originating in the southern portion of the bay^43–45^. Between 1 August and 25 September 2015 there was a bleaching event in Kāne'ohe Bay when seawater temperatures ranged from 28.4 to 29.8 °C^41^. During this period, corals from multiple reefs throughout Kāne'ohe Bay were impacted^41^, including those at reef K4. Throughout Kāne'ohe Bay, 16.6% ± 4.7 of *Montipora capitata* and 19.7% ± 0.7 of *Porites compressa* colonies bleached in 2015, with most colonies visibly recovered within 3–4 months of the event^46^.

In October 2015 when eggs started to develop^42^ and approximately one month after peak seawater temperatures^41^, we tagged visibly bleached and non-bleached colonies of both species at ~ 2 m depth at reef K4. Every 2–3 months colonies were monitored, and tags were cleaned to ensure the identification of the colonies for future collection prior to the spawning season when gametes were fully developed (Fig. 1). Both *M. capitata* and *P. compressa* have long gametogenic cycles that can last 9–10 months^42^ and coincide with the recovery of other physiological parameters of adult corals after a bleaching event^26^.

On 27 May 2016 (approximately eight months after the bleaching event), we collected fragments from four previously bleached and four previously non-bleached tagged colonies each of *M. capitata* and *P. compressa*. Colony fragments were approximately 10 cm^3^ each in size and contained at least eight fingers; similarly sized fragments were used from both species. At the time of collection, all colonies of both species appeared visibly non-bleached (Fig. 1); this was confirmed with no significant difference in chlorophyll *a* (µg/g) concentrations (method described in^26^) between previously bleached and non-bleached adult colonies (Student’s t-test for *M. capitata*: *df* = 3, F = 0.88, *p* = 0.4477; *P. compressa*: *df* = 7, F = 1.51, *p* = 0.2656). Coral fragments were transported to outdoor flow-through seawater tanks at the Hawai'i Institute of Marine Biology; the seawater was unfiltered, temperature ranged from 25.2 to 26.4 °C, and light availability ranged from 584 to 1249 µmol photons/m^2^/s. Each fragment was further divided into four nubbins for genetic replication across the following four treatments: autotrophy pulse-chase labeled; autotrophy unlabeled; heterotrophy pulse-chase labeled; and heterotrophy unlabeled.

On 1 June 2016 we began the autotrophy pulse-chase experiment. Autotrophy pulse-chase labeled and autotrophy unlabeled fragments were isolated in 2 L chambers filled with 0.2 µm-filtered and sterilized seawater to remove all plankton and limit heterotrophy of the nubbins. Chambers were placed in flow-through seawater tanks to maintain a constant ambient temperature through the experiment. One hour after sunrise, the pulse phase of the autotrophy experiment began with the introduction of 0.117 M of 98 at.% ^13^C NaHCO_3_ was added to each treatment chamber for a final concentration of dissolved inorganic carbon of approximately 26 µmol/l^25^. The same volume of unlabeled 0.2 µm-filtered and sterilized seawater was added to each control chamber. After 9 h, the isolation chambers were flushed with unlabeled 0.2 µm-filtered seawater to begin the chase phase. On day 1 (2 June 2016) and day 7 (8 June 2016) of the chase phase, we collected small pieces from each labeled and unlabeled colony for isotopic analyses. Water was exchanged within each chamber every 6 h during the chase period.

To prepare ^13^C-labelled rotifers for the heterotrophy pulse-chase experiment, we obtained cultures of native Hawaiian phytoplankton (*Nannochloropsis oculata*) and rotifers (*Brachionus plicatilis*). Cultures were maintained in 0.2 µm-filtered and sterilized seawater. The phytoplankton culture was grown with 0.117 M of 98 at.% ^13^C NaHCO_3_^25^ for at least two days and fed to the rotifer culture three times daily for three days prior to their use in the heterotrophy experiment. Resulting δ^13^C values for labeled and unlabeled phytoplankton were 75.82‰ and  −  15.87‰, respectively; δ^13^C values of labeled and unlabeled rotifers were 19.32‰ and  −  16.98‰, respectively. On 2 June 2016, heterotrophy pulse-chase labeled and heterotrophy unlabeled fragments were isolated in 2 L chambers filled with 0.2 µm-filtered and sterilized seawater to remove any non-labeled plankton from the chambers. One hour after sunset, the pulse phase of the heterotrophy experiment began with the introduction of 150 ml of ^13^C-labelled rotifers at a concentration of 2–4 rotifers per ml to each treatment chamber^25^. The same volume and concentration of unlabeled rotifers was added to each control chamber. Coral polyps were active and displayed extended tentacles. After eight hours, the isolation chambers were flushed with unlabeled unfiltered seawater to begin the chase phase. On day 1 (3 June 2016) and day 7 (9 June 2016) of the chase phase, we collected small pieces from each labeled and unlabeled colony for isotopic analyses. Coral samples from both pulse-chase experiments were collected and immediately frozen at -80 °C before being transported to Villanova University for further processing.

Coral tissue was removed from the skeleton using deionized water and an airbrush. Symbiont cells and host tissue were separated with a tissue grinder and centrifugation^47^. Separated cells and tissue were pipetted into different tin capsules (EA Consumables, LLC, Marlton, NJ), dried at 60 °C for at least 24 h. We visually checked all samples of symbiont cells and host tissues under a microscope prior to ensure that no skeletal pieces were in the capsules. Then, all capsules were folded into small, uniform pellets in preparation for isotopic analyses.

On the evenings of 5 and 6 June 2016, some *M. capitata* colonies released gamete bundles during a natural spawning event (1–2 days after the new moon). These dates coincided with days 3–4 of the chase phase for colonies in the heterotrophic experiment and days 4–5 of the chase phase for colonies in the autotrophic experiment (Fig. 1). Gamete bundles were collected from the surface of isolation chambers using pipettes for *M. capitata*^18^. We were unable to collect spawned eggs from *P. compressa*. This species has been sporadically observed to spawn during and after the full moon^38,48^ and the timing for gamete release is not as predictable as for *M. capitata*. To assess the isotopic signature of in situ developing eggs from both species, we preserved an additional fragment from each colony in 1.85% formaldehyde on day 7 of the chase. These fragments were decalcified using Cal-Ex II Fixative/Decalcifier, rinsed in 70% ethanol and developing eggs were dissected from the coral tissue. There was no significant difference in the isotopic values of gamete bundles released during the spawning event and eggs dissected from the same colony (paired t-test: *df* = 8, t = 0.25, *p* = 0.8090). Whether released or dissected, gametes bundles/eggs were pipetted into tin capsules, dried at 60 °C for at least 24 h, and folded into small, uniform pellets in preparation for isotopic analyses.

### Stable isotope analyses

All tin capsules were combusted in an Elementar Pyrocube and the resulting CO_2_ gas was analyzed with an Elementar Isoprime100 isotope ratio mass spectrometer at The Academy of Natural Sciences at Drexel University. δ^13^C values are reported relative to Vienna Peedee Belemnite Limestone Standard (vPDB) (δ^13^C = per mil deviation of the ratio of stable carbon ^13^C:^12^C relative to vPDB). Samples were analyzed in duplicate. Standards, B2150 (EA Consumables, LLC, Marlton, NJ), internal elk tissue, DORM (fish muscle) and bird feather standards had a precision of ± 0.14‰ for δ^13^C.

### Statistical analyses

Statistically significant differences in δ^13^C values were determined separately for each species and trophic pulse-chase experiment with mixed effects modeling (Supplementary Table 1). These compared the effects of prior bleaching status (bleached, non-bleached), pulse period treatment (^13^C-labeled, unlabeled), and tissue type (symbiont, host, eggs/bundles), and the repeated effect of time during the chase period (day 1, day 7). Each model included a random effect of genotype and tissue type (symbiont cells, host tissue and egg/bundle) was nested within prior bleaching status. The random and repeated effects were compared with covariance parameter estimates and the fit statistic, -2res log likelihood. Post-hoc Tukey–Kramer tests determined the factors that were significantly different from each other within significant interactions of the main model effects (Supplementary Tables 2–5). *p* ≤ 0.05 was considered statistically significant. We calculated percent enrichment values to compare average labeled values to their respective controls for each pulse-chase experiment and species (Supplementary Table 6). All statistical analyses were generated using SAS statistical software Version 9.4 of the SAS System for Windows.

## Results and discussion

### Pathways for autotrophic acquisition of carbon

**Figure 2:**
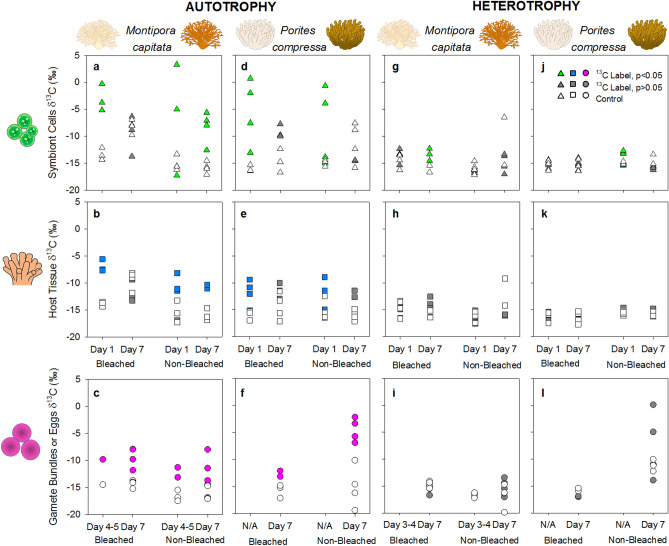
Carbon acquisition for gamete development is predominantly autotrophic. (**a**–**l**) Acquisition of carbon by autotrophy or heterotrophy in *Montipora capitata* (**a**–**c, g**–**i**) and *Porites compressa* (**d**–**f, j**–**l**) colonies. Allocation occurred during the chase phase to symbiont cells (**a, d, g, j**), host tissue (**b, e, h, k**), and eggs (**c, f, i, l**) for each species. All stable carbon isotopic values (δ^13^C) are shown; with control colonies (DI^12^C or ^12^C-rotifers) in white symbols; labeled colonies (DI^13^C or ^13^C-rotifers) in colored symbionts. Green, blue, or pink symbols indicate statistically significant differences compared to control colonies; and labeled colonies in grey symbols indicate no significant difference compared to control colonies. Complete statistical analyses can be found in Supplementary Tables 1–5.

Our results provide the first direct evidence of resource allocation of carbon from adult coral colonies to their eggs (Fig. 2). Both species relied on autotrophy for carbon allocation to eggs. By day 1 of the chase for *Montipora capitata*, there was significant incorporation of ^13^C from autotrophy in symbiont cells (Fig. 2a) and host tissue (Fig. 2b) in labeled compared to control corals of both bleached and non-bleached adult colonies. Similarly, by days 4–5 there was significant ^13^C enrichment in labeled compared to control gamete bundles released from both bleached and non-bleached adult colonies (Fig. 2c). In *M. capitata*, the location of ^13^C allocation and its retention time within adults during the chase phase was different based on prior bleaching status. In bleached colonies, significantly more ^13^C was allocated to symbiont cells than host tissue, and significantly more ^13^C was allocated to host tissue than gamete bundles beginning on day 1 of the chase (Fig. 2a–c; Supplementary Table 2). By day 7, ^13^C was depleted (i.e., lower δ^13^C) in both symbiont cells and host tissue of bleached corals with no significant difference between labeled and control colonies (Fig. 2a, b). However, eggs of bleached colonies continued to be a significant storage site (i.e., higher δ^13^C) for ^13^C allocation at day 7 (Fig. 2c). In contrast, non-bleached colonies retained ^13^C-label in symbiont cells, host tissue, and eggs through day 7 of the chase (Fig. 2a–c). Despite this, there was significantly more ^13^C translocated to developing eggs of bleached than non-bleached colonies by day 7 (Fig. 2c; Supplementary Table 2). Altogether these patterns indicate significant prioritization on gamete development over adult tissue maintenance in *M. capitata* when carbon is autotrophically-acquired, particularly when bleaching has occurred.

For both bleached and non-bleached *Porites compressa*, patterns of autotrophic allocation of ^13^C were similar to that of bleached *M. capitata*. In *P. compressa* on day 1 of the chase, there was significantly greater incorporation of ^13^C in symbiont cells (Fig. 2d) and host tissue (Fig. 2e) in labeled compared to control corals of both bleached and non-bleached adult colonies (Supplementary Table 3). There was more ^13^C allocated to the symbiont than the host regardless of prior bleaching status. By day 7, ^13^C-label was significantly depleted in symbiont cells and host tissue of both bleached and non-bleached adult colonies (Fig. 2d, e; Supplementary Table 3). Although we were unable to assess the gametes of *P. compressa* before day 7 of the chase, eggs dissected from both bleached and non-bleached colonies were significantly more enriched in ^13^C in labeled compared to control corals (Fig. 2f). In contrast to *M. capitata*, there was greater allocation of ^13^C to eggs of non-bleached than bleached colonies in *P. compressa* (Fig. 2f; Supplementary Table 3). Adults that did not bleach were able to allocate more autotrophically-acquired carbon to their eggs than those that did bleach.

### Pathways for heterotrophic acquisition of carbon

While prior biochemical studies of adult coral colonies hypothesized that heterotrophy would be critical to egg development in *M. capitata* especially following bleaching and less so in *P. compressa*^26,28^, we find that neither species relied heavily on heterotrophy. Approximately 8 months after a natural bleaching event, resource allocation of both species to their eggs relied predominantly on autotrophically-acquired carbon (Fig. 2). In *M. capitata*, there was little acquisition of heterotrophic ^13^C by symbiont cells (Fig. 2g) and host tissue (Fig. 2h) of adult colonies and developing eggs or released gamete bundles (Fig. 2i), except by symbiont cells from bleached colonies at day 7 (Fig. 2g; Supplementary Table 4). Although *M. capitata* has been shown to feed more heterotrophically while bleached and in the early months of recovery from bleaching^47,49,50^, it is more reliant on autotrophy than other conspecifics when non-bleached^28^. More recently, δ^13^C analyses indicated a limited influence of heterotrophy on *M. capitata* during post-bleaching recovery^51^. Our data indicate that carbon from heterotrophy was not incorporated by gametes in *M. capitata*, suggesting that this source is less critical to gametogenesis than previously hypothesized.

In contrast, heterotrophic carbon was incorporated into the developing eggs of some non-bleached colonies of *P. compressa*, although not statistically significant as a group (Fig. 2l). Similarly, *P. compressa* adults showed little to no acquisition of heterotrophic ^13^C, except by day 1 when there was significant enrichment of ^13^C by symbiont cells from non-bleached colonies (Fig. 2j; Supplementary Table 5). This indicates that if feeding occurred during the experiment, any associated carbon was metabolized quickly (within hours) of the chase. By day 7, the developing eggs of non-bleached labeled *P. compressa* were on average 40% more enriched with ^13^C than non-bleached control colonies (Fig. 2l). Like autotrophic acquisition, there was greater allocation of ^13^C to eggs from non-bleached to bleached colonies of *P. compressa* following heterotrophy (Fig. 2l; Supplementary Table 5). Accumulation of heterotrophic carbon in *P. compressa* eggs of some colonies at day 7 (Fig. 2l), while not statistically significant, may have been translocated via the host tissue in less than 24 h. Transfer of carbon is known to occur within 24 h between cellular organelles of adult tissues^52^ and among tissue layers of larvae^53^, therefore translocation from parent to gametes may also occur under similar time periods. Heterotrophic acquisition of carbon to eggs may occur in *P. compressa* and deserves further investigation.

Low enrichment values indicate limited evidence of carbon allocation by heterotrophy. This may be due to loss of carbon via respiration of the coral holobiont^25^ and/or mucus production^54^ and subsequent uptake of carbon by microbes in coral mucus^55^. The timing of the experiment with respect to spawning periods of each species, may have further impacted carbon uptake by heterotrophy. For *M. capitata*, spawning occurred in the midst of the experiment, while for *P. compressa* spawning would have occurred approximately two weeks later ^38,48^. It is important to note that some colonies of *P. compressa* contained eggs with heterotrophically-acquired carbon, suggesting that coral feeding can contribute to egg development in this species. To date we know little about how or if coral feeding behavior changes as eggs grow and occupy more space in the gastrovascular cavity or how feeding is impacted by the process of bundle formation and preparation for gamete release. Further research is needed to better understand the role of heterotrophy in coral reproduction^34,35^.

### Parental provisioning of carbon

Parental provisioning in corals with different reproductive strategies (brooders vs spawners) may occur at different rates and stages during gametogenesis and/or embryogenesis. In our study we found that broadcast spawners transferred carbon mostly autotrophically to eggs within days before their release. Similarly, gametes of *M. capitata*^56^ and *P. compressa*^48^ both receive symbionts from their parents prior to their release (i.e., vertical transmission), not from the environment (i.e., horizontal transmission). In the brooding coral, *Stylophora pistillata* translocation of carbon preferentially occurred at the planula (post-fertilization) stage^36^, while planulae of *S. pistillata* are known to receive symbionts both by vertical and horizontal transmission^57^. Regardless of trophic source, carbon allocation from parents to gametes may coincide with vertical transmission of symbionts to more effectively supply offspring with resources required immediately upon release.

Pulse-chase experiments conducted on planulae support this hypothesis. For example, planulae of *Pocillopora damicornis* relied on carbon supplied by the parent for at least 24 h after being released, then shifted to carbon acquired autotrophically from their own symbionts at 48 h^53^. Progressively older larvae relied more on autotrophy for their carbon supply than on parental reserves. *Pocillopora damicornis* planulae < 5 days old received 16–27% of fixed carbon from their symbionts^58^, while nearly one-month old planulae acquired approximately 70–85% of carbon from symbionts^59,60^. Similarly-aged *Montipora digitata* larvae acquired up to 90% of fixed carbon from photosynthate of their symbionts^60^. Although it is not known when or by what trophic pathway parental provisioning occurs in *P. damicornis* (a brooding species with vertical transmission) or *M. digitata* (a spawning species with vertical transmission), parental sources of carbon are relied upon immediately once offspring reach the water column and for several subsequent days. The initial lack of photosynthate production by symbionts in gametes/larvae of these species may prevent oxidative damage during the first 24 h of release as they drift near the surface and may be at greater risk of heat stress^61^. Thus, further emphasizing the importance of parental provisioning of carbon to offspring.

### Parental trade-offs

Trade-off theory indicates that reproduction will generally interfere with maintenance and/or growth of the parent, with metabolic trade-offs becoming more complex when symbiosis is involved^62^. For example, in damaged or dislodged coral colonies, metabolic costs associated with regeneration resulted in decreased reproduction^63,64^. In our study, we found that coral parents translocated carbon resources to their gametes prior to release, but this occurred at a detriment to carbon storage in adult tissue. Adult colonies of both species experienced a physiological trade-off when supplying their eggs with carbon, especially among those colonies that had previously bleached. Gametes enriched in ^13^C coincided with depletion of ^13^C in both symbiont cells and host tissue, evidence of a resource trade-off for parents. Loss of carbon from the parent tissue due to reproduction is expected and has been calculated as part of carbon budgets^36,65^, or implicated from field observations and energy reserve analyses^15^. This is the first direct measure of that metabolic trade-off as carbon depletion in parental tissues.

In our study, we cannot assess how much ^13^C, if any, was lost by adult tissues through respiration. However, no change in ^13^C between day 1 and 7 in non-bleached symbiont cells (Fig. 2a) or host tissue (Fig. 2b) of *M. capitata* (Supplementary Table 2), suggests that respiration of newly acquired carbon was minimal. A trade-off in carbon allocation occurred within just 7 days of acquisition in our study, suggesting that maintenance of symbiont cells and host tissue are being impacted by carbon requirements for developing gametes over relatively short time periods. In the case of *M. capitata* this trade-off occurred for carbon acquired autotrophically by previously bleached colonies only, while for *P. compressa* autotrophically-acquired carbon was translocated from adults to developing eggs by both bleached and non-bleached colonies. Reallocation of carbon away from adults and for egg development occurred in both species.

Other studies have shown long-term trade-offs for adult colonies that reproduce. For example, reproducing colonies of *P. damicornis* had half the annual linear extension rate of non-reproducing colonies^65^. Long-term trade-offs have also been associated with reproduction following a bleaching event. In *Orbicella annularis* (previously *Montastraea annularis*), gametogenesis occurred after bleaching in colonies that had visibly recovered, while colonies that remained visibly bleached consumed their own structural material for maintenance and did not reproduce^15,17^. In *Acropora* spp. reproductive output was proportional to bleaching susceptibility, but growth rates were independent of bleaching severity^66^. While bleaching is known to reduce calcification in some Hawaiian species^9,47^, reproduction continued despite bleaching^18^. To date, long-term trade-offs associated with growth and reproduction after a bleaching event remain unclear^67^ and warrant further investigation.

### Trophic dynamics and coral metabolism

Heterotrophy in bleached corals is hypothesized to be indicative of a colony under stress and/or a resilience mechanism to survive bleaching^50,68^. Adult reliance on heterotrophic acquisition of ^13^C was lower in our study (i.e., eight months after natural bleaching) compared to eleven months after experimental bleaching for the same species^50^. Differences in adult response were probably due to differences in temperature severity as the experimental bleaching was ~ 3 °C warmer than the 2015 natural bleaching event of our study^25,69^. In addition, shading, cloud cover, rain events, and flow may all moderate conditions on a reef during some natural bleaching events, allowing affected corals to recover faster^69^ than with more controlled temperature treatments during experimental bleaching. Our findings suggest that the duration of heterotrophic reliance post-bleaching depends on the severity of the stress.

While heterotrophic acquisition of carbon seems indicative of bleaching severity in some coral species^50,70^, autotrophic acquisition and allocation may be more indicative of the physiological stress on the adult colony. In previously bleached colonies of *M. capitata* and all colonies of *P. compressa*, autotrophically-acquired ^13^C was catabolized, not stored, as it decreased from day 1 to day 7 of the chase (Fig. 2a–f). Conversely, newly acquired carbon was stored and maintained in colonies of *M. capitata* that did not bleach in the 2015 event. Our results show that even if bleached *M. capitata* colonies recovered (i.e., acquired symbionts and developed gametes), there was still a fundamental difference in carbon requirements between bleached and non-bleached colonies eight months later.

Species-specific differences in how carbon is allocated following bleaching may reflect differences in the timing of their gametogenic cycles and spawning periods. In Hawai'i, the timing between the bleaching season (Sept-Oct) and the spawning season (May–June) is at least seven months^41,71^. Recovery of bleached colonies and the development of gametes can occur within this timeframe. In *M. capitata*, egg development starts in Aug-Sept, takes approximately eight to ten months and spawning occurs during May-Aug, two to four days after the new moon^18,42^. The length of the gametogenic cycle in *P. compressa* is unknown, but sporadic observations indicate that this species releases gametes during and after the full moon in summer months^38,48^. In our experiments, *M. capitata* had already spawned on days 3–5 of the chase, while *P. compressa* may have spawned ~ 11–12 days after the conclusion of our study. Significant translocation of ^13^C from adults to in situ eggs may indicate that *P. compressa* was allocating resources to eggs and preparing for spawning (Fig. 2f, l). Although we were unable to observe spawning in *P. compressa*, our data clearly show that this species has the capacity to produce gametes after a bleaching event and that carbon was supplied to eggs in both previously bleached and non-bleached colonies. For both species, long-term recovery of bleached colonies occurred in parallel to the development of gametes^42^. As thermal stress events become more frequent, intense and longer in duration, the window to recover and develop gametes may shorten, impacting reproductive life history strategies.

### Strategies for reproduction following coral bleaching

A comparison of previously bleached and non-bleached colonies provides evidence of the physiological impacts that occur eight months after a natural bleaching event in adults and developing gametes. Previously bleached *M. capitata* depleted carbon storage within symbiont cells (by 80%) and host tissue (by 50%) compared to non-bleached colonies (Fig. 2a, b). Yet, translocating carbon to eggs was an energetic priority for both bleached and non-bleached colonies (Fig. 2c). Carbon translocation to eggs occurred at the detriment of bleached adult colonies, while carbon storage was maintained in non-bleached adult colonies. Furthermore, previously bleached colonies supplied more ^13^C to eggs (by 10%) at day 7 than non-bleached colonies (Fig. 2c, Supplementary Table 2). These findings show that the *M. capitata* prioritized gamete development and that the amount of energy supplied by the parent for this physiological process is not limited by prior bleaching. Previously bleached colonies were able to meet the energy demand for eggs by depleting stored carbon reserves in adults. These results support those by Cox^18^ and provide a mechanism for continued gametogenesis in *M. capitata* despite bleaching. Additionally, similar amounts of carbon were translocated to developing eggs despite differences in bleaching susceptibility (Supplementary Table 2). This supports previous findings that identified low phenotypic and biochemical variability of eggs in *M. capitata* from parents with distinct morphology, physiology, and exposure to environmental stress^72^.

In *P. compressa*, gametogenesis continued after bleaching and developing eggs were provisioned with newly acquired carbon in previously bleached and non-bleached colonies. Although ^13^C was incorporated autotrophically to eggs in *P. compressa* (Fig. 2f, l), there was no difference in incorporation of carbon by symbiont cells or host tissue in bleached compared to non-bleached colonies via either trophic pathway (Supplementary Tables 3, 5). This suggests that *P. compressa* adults had physiologically recovered from the bleaching event^26^. Yet, we observed at least one remaining impact of the bleaching event in their developing eggs, as 45–80% less carbon was translocated to eggs of previously bleached than non-bleached colonies (Fig. 2f, l). *Porites compressa* may only supply carbon to eggs when there is surplus available and after the adult has recovered. While bleached *P. compressa* produced eggs and may spawn after a bleaching event, non-bleached colonies likely spawn earlier or produce more, or larger eggs compared to bleached colonies.

Our study highlights two different strategies of coral parental provisioning with important consequences for the survival of adults and offspring in the context of future and repeated bleaching events. By increasing carbon allocated to eggs, bleached *M. capitata* prioritized gametogenesis at the expense of the adult colony. Higher investment in reproduction may help compensate for the low survival of early recruits of this species^40^. For *P. compressa*, there was no difference in the allocation of carbon to the host tissues of bleached and non-bleached adult colonies. However, less carbon was transferred to eggs of bleached colonies, and this may lead to smaller and/or fewer eggs in bleached colonies. Since *P. compressa* has less evolutionary pressure to produce large amounts of gametes^40^, this strategy for carbon allocation may maintain reproductive potential after a bleaching event.

## Supplementary Information

Supplementary Information.

## Data Availability

All data are provided in the main manuscript and in the supplemental material.
