# Supplementary material for: Trophic provisioning and parental trade-offs lead to successful reproductive performance in corals after a bleaching event
Source: Sci Rep. 2022 Nov 4;12:18702. doi: 10.1038/s41598-022-21998-4 (PMC9636168; doi:10.1038/s41598-022-21998-4)
Supplement: Supplementary file 1 — Supplementary Information. [file 41598_2022_21998_MOESM1_ESM.pdf]

Supplementary Table 1. Results of mixed effects modeling for $\delta^{13}C$ of each species and trophic pathway.

	Montipora capitata			*Porites compressa*		
**Autotrophy Pulse-Chase Experiments**						
**Fixed Effects**	**df[1]**	**F Value**	**p-value[2]**	**df[1]**	**F Value**	**p-value[2]**
Bleaching Status (B)[3]	1, 56	7.30	**0.0091**	1, 41	0.08	0.7751
Pulse Treatment (P)[4]	1, 56	110.63	**<0.0001**	1, 41	68.89	**<0.0001**
Tissue (T)[5]	2, 56	11.71	**<0.0001**	2, 41	6.21	**0.0044**
Day (D)[6]	1, 56	0.34	0.5642	1, 41	0.83	0.3689
B x P	1, 56	3.64	0.0617	1, 41	0.01	0.9393
B x T	2, 56	2.59	0.0843	2, 41	6.96	**0.0025**
B x D	1, 56	0.56	0.4582	1, 41	0.74	0.3956
P x T	2, 56	4.03	**0.0232**	2, 41	7.31	**0.0019**
P x D	1, 56	15.78	**0.0002**	1, 41	2.42	0.1272
T x D	2, 56	0.31	0.7355	1, 41	1.05	0.3105
B x P x T	2, 56	1.32	0.2754	2, 41	3.72	**0.0326**
B x P x D	1, 56	11.62	**0.0012**	1, 41	0.27	0.6045
B x T x D	2, 56	0.34	0.7143	1, 41	0.15	0.6997
P x T x D	2, 56	5.50	**0.0066**	1, 41	0.67	0.4170
B x P x T x D	2, 56	6.44	**0.0030**	1, 41	0.17	0.6839
**Variance Estimates**	**df[1]**	**Estimate[7]**	**p-value[8]**	**df[1]**	**Estimate[7]**	**p-value[8]**
Genotype[9]	1	1.5350	**<0.0001**	1	1.8300	**0.0200**
Tissue (Bleaching Status)	5	4.4434	**0.0002**	5	6.8729	**0.0300**
**Heterotrophy Pulse-Chase Experiments**						
**Effect**	**df[1]**	**F Value**	**p-value[2]**	**df[1]**	**F Value**	**p-value[2]**
Bleaching Status (B)[3]	1, 49	1.23	0.2727	1, 45	19.34	**<0.0001**
Pulse Treatment (P)[4]	1, 49	0.84	0.3627	1, 45	1.52	0.2243
Tissue (T)[5]	2, 49	1.814	0.1740	2, 45	20.72	**<0.0001**
Day (D)[6]	1, 49	4.56	**0.0377**	1, 45	0.80	0.3760
B x P	1, 49	3.30	0.0755	1, 45	1.66	0.2040
B x T	2, 49	4.56	**0.0153**	2, 45	8.73	**0.0006**
B x D	1, 49	13.69	**0.0005**	1, 45	0.80	0.3747
P x T	2, 49	0.14	0.8706	2, 45	0.79	0.4591
P x D	1, 49	0.21	0.6455	1, 45	7.03	**0.0110**
T x D	2, 49	2.33	0.1079	1, 45	0.55	0.4621
B x P x T	2, 49	10.98	**0.0001**	2, 45	2.05	0.1408
B x P x D	1, 49	7.65	**0.0080**	1, 45	8.30	**0.0060**
B x T x D	1, 49	0.11	0.7364	1, 45	0.72	0.3993
P x T x D	2, 49	3.33	**0.0440**	1, 45	4.30	**0.0439**
B x P x T x D	1, 49	0.01	0.9426	1, 45	4.89	**0.0321**
**Variance Estimates**	**df[1]**	**Estimate[7]**	**p-value[8]**	**df[1]**	**Estimate[7]**	**p-value[8]**
Genotype[9]	1	0.1658	0.1600	1	0.1975	**0.0080**
Tissue (Bleaching Status)	5	2.1738	**0.0400**	5	3.3563	**<0.0001**

[1]Degrees of freedom (df) are shown for each effect by numerator, denominator

[2]Statistically significant p-values < 0.05 are shown in bold
[3]Prior bleaching status (previously bleached, previously non-bleached), nested with tissue type
[4]Pulse treatment (^{13}C-labelled, unlabeled)
[5]Tissue type (symbiont, host, eggs/bundles), nested with prior bleaching status
[6]Day during the chase period (day 1, day 7), repeated effect
[7]Variances estimates for random and repeated effects in the model
[8]Based on the difference in fit statistics (-2res log likelihood) of the model
[9]Genotype (four independent genotypes per species), random effect

Supplementary Table 2. Post-hoc Tukey-Kramer comparisons for $\delta^{13}C$ of *Montipora capitata* following autotrophy pulse-chase experiments. For each pairwise comparison, statistically significant differences (p > 0.05) are shown in bold and the relationship of the comparison: greater than (>), less than (<), or equal to (=) is indicated.

Day	Status	Pulse	Tissue	p-value
1	Bleached (B)	Labeled (L)	**S > H**	**0.0057**
			**S > G**	**<0.0001**
			**H > G**	**0.0137**
		**L > U**	Symbiont (S)	**<0.0001**
		**L > U**	Host (H)	**<0.0001**
		**L > U**	Gamete Bundles (G)	**0.0022**
		Unlabeled (U)	S=H	0.8121
			S=G	0.4091
			H=G	0.4373
	B=NB	Labeled	Symbiont	0.2661
	**B > NB**		Host	**0.0005**
	B=NB		Gamete Bundles	0.5189
	B=NB	Unlabeled	Symbiont	0.5124
	B=NB		Host	0.1312
	B=NB		Gamete Bundles	0.3927
	Non-Bleached (NB)	Labeled	**S > H**	**0.0197**
			**S > G**	**0.0397**
			H=G	0.4928
		**L > U**	Symbiont	**0.0041**
		**L > U**	Host	**0.0011**
		**L > U**	Gamete Bundles	**<0.0001**
		Unlabeled	S=H	0.7345
			S=G	0.6354
			H=G	0.8003
**1 > 7**	Bleached	Labeled	Symbiont	**0.0004**
**1 > 7**			Host	**<0.0001**
1=7			Gamete Bundles	0.5200
**1 < 7**		Unlabeled	Symbiont	**0.0004**
**1 < 7**			Host	**<0.0001**
1=7			Gamete Bundles	0.6471
1=7	Non-Bleached	Labeled	Symbiont	0.4744
1=7			Host	0.8254
1=7			Gamete Bundles	0.4946
1=7		Unlabeled	Symbiont	0.8187
1=7			Host	0.8819
1=7			Gamete Bundles	0.7885
7	Bleached	Labeled	S=H	0.1198

			S=G	0.9716
			**H < G**	**0.0169**
		L=U	Symbiont	0.4566
		**L < U**	Host	**0.0227**
		**L > U**	Gamete Bundles	**<0.0001**
		Unlabeled	S=H	0.2920
			**S > G**	**<0.0001**
			**H > G**	**<0.0001**
	B=NB	Labeled	Symbiont	0.6795
	B=NB		Host	0.8579
	**B > NB**		Gamete Bundles	**0.0468**
	**B > NB**	Unlabeled	Symbiont	**0.0022**
	**B > NB**		Host	**<0.0001**
	B=NB		Gamete Bundles	0.0895
	Non-Bleached	Labeled	S=H	0.1179
			S=G	0.0926
			H=G	0.8511
		**L > U**	Symbiont	**0.0095**
		**L > U**	Host	**0.0003**
		**L > U**	Gamete Bundles	**<0.0001**
		Unlabeled	S=H	0.9054
			S=G	0.7720
			H=G	0.7421

Supplementary Table 3. Post-hoc Tukey-Kramer comparisons for $\delta^{13}C$ of *Porites compressa* following autotrophy pulse-chase experiments (no data for Day 1 eggs, bleached or non-bleached). For each pairwise comparison, statistically significant differences (p > 0.05) are shown in bold and the relationship of the comparison: greater than (>), less than (<), or equal to (=) is indicated.

Day	Status	Pulse	Tissue	p-value
1	Bleached (B)	Labeled (L)	**S > H**	**0.0033**
		**L > U**	Symbiont (S)	**0.0003**
		**L > U**	Host (H)	**0.0114**
		Unlabeled (U)	S=H	0.9710
	B=NB	Labeled	Symbiont	0.1629
	B=NB		Host	0.2890
	B=NB	Unlabeled	Symbiont	0.7183
	B=NB		Host	0.9749
	Non-Bleached (NB)	Labeled	**S > H**	**0.0330**
		**L > U**	Symbiont	**0.0376**
		**L > U**	Host	**0.0090**
		Unlabeled	S=H	0.6622
1=7	Bleached	Labeled	Symbiont	0.1798
1=7			Host	0.8118
1=7		Unlabeled	Symbiont	0.6209
1=7			Host	0.2952
1=7	Non-Bleached	Labeled	Symbiont	0.2525
1=7			Host	0.1985
1=7		Unlabeled	Symbiont	0.7978
1=7			Host	0.7011
7	Bleached	Labeled	S=H	0.3317
			S=E	0.1299
			H=E	0.4607
		L=U	Symbiont	0.0578
		L=U	Host	0.0734
		**L > U**	Eggs (E)	**0.0039**
		Unlabeled	**S=H**	0.8725
			**S=E**	0.5904
			**H=E**	0.2339
	B=NB	Labeled	Symbiont	0.2805
	**B > NB**		Host	**0.0378**
	**B < NB**		Eggs	**0.0005**
	B=NB	Unlabeled	Symbiont	0.7198
	B=NB		Host	0.1887
	B=NB		Eggs	0.9128

Non-Bleached	Labeled	S=H	0.1790
		**S < E**	**0.0068**
		**H < E**	**<0.0001**
	L=U	Symbiont	0.1925
	L=U	Host	0.1445
	**L > U**	Eggs	**<0.0001**
	Unlabeled	S=H	0.7840
		S=E	0.9172
		H=E	0.8365

Supplementary Table 4. Post-hoc Tukey-Kramer comparisons for $\delta^{13}C$ of *Montipora capitata* following heterotrophy pulse-chase experiments. For each pairwise comparison, statistically significant differences ($p > 0.05$) are shown in bold and the relationship of the comparison: greater than (>), less than (<), or equal to (=) is indicated.

Day	Status	Pulse	Tissue	p-value
1	Bleached (B)	Labeled (L)	S=H	0.1058
		L=U	Symbiont (S)	0.1540
		L=U	Host (H)	0.6552
		Unlabeled (U)	S=H	0.8034
	B=NB	Labeled	Symbiont	0.0788
	B=NB		Host	0.1661
	B=NB	Unlabeled	Symbiont	0.4108
	B=NB		Host	0.1034
	Non-Bleached (NB)	Labeled	S=H	0.9754
			S=G	0.8053
			H=G	0.7394
		L=U	Symbiont	0.8181
		L=U	Host	0.9045
		L=U	Gamete Bundles (G)	0.8955
		Unlabeled	S=H	0.8106
			S=G	0.6204
			H=G	0.7384
**1=7**	Bleached	Labeled	Symbiont	0.8825
1=7			Host	0.5214
1=7		Unlabeled	Symbiont	0.1179
1=7			Host	0.1753
1=7	Non-Bleached	Labeled	Symbiont	0.4501
1=7			Host	0.3686
1=7			Gamete Bundles	0.0634
**1 < 7**		Unlabeled	Symbiont	**0.0239**
**1 < 7**			Host	**0.0018**
1=7			Gamete Bundles	0.8835
7	Bleached	Labeled	S=H	0.3114
			**S > G**	**0.0087**
			H=G	0.1574
		**L > U**	Symbiont	**0.0049**
		L=U	Host	0.1313
		L=U	Gamete Bundles	0.1101
		Unlabeled	S=H	0.8127
			**S < G**	**0.0437**
			H=G	0.1384
	B=NB	Labeled	Symbiont	0.3026

B=NB		Host	0.4082
B=NB		Gamete Bundles	0.6590
**B < NB**	Unlabeled	Symbiont	**0.0075**
**B < NB**		Host	**0.0052**
**B > NB**		Gamete Bundles	**0.0096**
Non-Bleached	Labeled	S=H	0.7993
		S=G	0.9367
		H=G	0.8049
	L=U	Symbiont	0.0683
	**L < U**	Host	**0.0177**
	L=U	Gamete Bundles	0.0720
	Unlabeled	S=H	0.6963
		**S > G**	**0.0031**
		**H > G**	**0.0003**

Supplementary Table 5. Post-hoc Tukey-Kramer comparisons for $\delta^{13}C$ of *Porites compressa* following heterotrophy pulse-chase experiments (no data for Day 1 eggs, bleached or non-bleached). For each pairwise comparison, statistically significant differences ($p > 0.05$) are shown in bold and the relationship of the comparison: greater than (>), less than (<), or equal to (=) is indicated.

Day	Status	Pulse	Tissue	p-value
1	Bleached (B)	Labeled (L)	S=H	0.1550
		L=U	Symbiont (S)	0.7327
		L=U	Host (H)	0.3304
		Unlabeled (U)	S=H	0.0831
	**B < NB**	Labeled	Symbiont	**0.0105**
	B=NB		Host	0.3041
	B=NB	Unlabeled	Symbiont	0.5825
	B=NB		Host	0.1712
	Non-Bleached (NB)	Labeled	**S > H**	**<0.0001**
		**L > U**	Symbiont	**0.0061**
		L=U	Host	0.3681
		Unlabeled	S=H	0.1444
1=7	Bleached	Labeled	Symbiont	0.8744
1=7			Host	0.9963
1=7		Unlabeled	Symbiont	0.9378
1=7			Host	0.9448
**1 > 7**	Non-Bleached	Labeled	Symbiont	**<0.0001**
1=7			Host	0.3614
1=7		Unlabeled	Symbiont	0.0830
1=7			Host	0.6064
7	Bleached	Labeled	S=H	0.1122
			**S > E**	**0.0009**
			**H > E**	**0.0429**
		L=U	Symbiont	0.5399
		L=U	Host	0.2595
		**L < U**	Eggs (E)	**0.0232**
		Unlabeled	S=H	0.0487
			S=E	0.6543
			H=E	0.1486
	B=NB	Labeled	Symbiont	0.2296
	B=NB		Host	0.6088
	**B < NB**		Eggs	**<0.0001**
	**B < NB**	Unlabeled	Symbiont	**0.0388**
	B=NB		Host	0.0834
	**B < NB**		Eggs	**0.0451**

Non-Bleached	Labeled	S=H	0.6188
		**S < E**	**0.0003**
		**H < E**	**0.0004**
	**L < U**	Symbiont	**0.0035**
	L=U	Host	0.5993
	L=U	Eggs	0.2255
	Unlabeled	**S > H**	**0.0114**
		S=E	0.2043
		H=E	0.0519

Supplementary Table 6. Average enrichment (%) values and standard deviation (SD) for labeled autotrophy and heterotrophy pulse-chase samples relative to their respective control samples in *Montipora capitata* and *Porites compressa*. na = sample sizes too small to calculate SD; nd = no data

			Montipora capitata		*Porites compressa*	
**Autotrophy Pulse-Chase Experiments**						
**Tissue**	**Day**	**Status**	**Enrichment (%)**	**SD (%)**	**Enrichment (%)**	**SD (%)**
Symbiont	1	Bleached	126.66	1.09	98.22	2.18
Symbiont	7	Bleached	-16.46	1.59	45.61	1.03
Symbiont	1	Non-Bleached	82.32	4.24	56.42	2.54
Symbiont	7	Non-Bleached	62.26	1.12	26.06	1.32
Host	1	Bleached	66.97	0.49	29.36	0.94
Host	7	Bleached	-22.22	1.04	22.64	1.05
Host	1	Non-Bleached	28.34	1.46	18.95	1.22
Host	7	Non-Bleached	31.49	0.80	9.21	0.73
Gamete Bundles or Eggs	4-5	Bleached	38.44	na	nd	nd
Gamete Bundles or Eggs	7	Bleached	41.46	0.70	21.75	0.63
Gamete Bundles or Eggs	4-5	Non-Bleached	25.12	0.53	nd	nd
Gamete Bundles or Eggs	7	Non-Bleached	31.95	1.10	107.59	1.56

**Heterotrophy Pulse-Chase Experiments**						
**Tissue**	**Day**	**Status**	**Enrichment (%)**	**SD (%)**	**Enrichment (%)**	**SD (%)**
Symbiont	1	Bleached	8.11	0.72	0.95	0.45
Symbiont	7	Bleached	17.93	0.64	2.03	0.47
Symbiont	1	Non-Bleached	-2.94	0.40	9.84	0.46
Symbiont	7	Non-Bleached	-31.00	3.19	-10.36	0.63
Host	1	Bleached	-2.67	0.68	2.66	0.46
Host	7	Bleached	9.22	0.72	3.11	0.45

Host	1	Non-Bleached	-0.81	0.47	1.63	0.26
Host	7	Non-Bleached	-26.90	1.82	0.79	0.35
Gamete Bundles or Eggs	3-4	Bleached	nd	nd	nd	nd
Gamete Bundles or Eggs	7	Bleached	-5.25	0.40	-8.07	0.19
Gamete Bundles or Eggs	3-4	Non-Bleached	-2.55	0.16	nd	nd
Gamete Bundles or Eggs	7	Non-Bleached	10.67	0.95	40.12	2.22

Supplementary Table 7. Raw data of δ^{13}C values presented in Fig. 2 of the manuscript, for each day, species (*Montipora capitata*, MC or *Porites compressa*, PC), status (bleached, B or non-bleached, NB), isotopic label (autotrophy, Auto and control autotrophy, Con. Auto, heterotrophy, Hetero and control heterotrophy, Con. Hetero), and tissue type (host, symbiont, Symb, eggs/bundles).

Day	Spp	Status	ID	Label	Tissue	δ^{13}C
1	MC	B	127	Auto	Host	-7.47746
1	MC	B	131	Auto	Host	-5.58225
1	MC	B	139	Auto	Host	-7.63907
1	MC	B	127	Con. Auto	Host	-13.4923
1	MC	B	131	Con. Auto	Host	-13.7673
1	MC	B	139	Con. Auto	Host	-14.4004
1	MC	B	141	Con. Auto	Host	-13.7225
1	MC	B	127	Con. Hetero	Host	-13.3407
1	MC	B	131	Con. Hetero	Host	-16.6601
1	MC	B	139	Con. Hetero	Host	-14.7071
1	MC	B	141	Con. Hetero	Host	-13.555
1	MC	B	127	Hetero	Host	-13.6537
1	MC	B	131	Hetero	Host	-16.5146
1	MC	B	139	Hetero	Host	-14.9295
1	MC	B	141	Hetero	Host	-14.7394
1	MC	NB	126	Auto	Host	-11.4777
1	MC	NB	130	Auto	Host	-8.18277
1	MC	NB	138	Auto	Host	-16.9374
1	MC	NB	140	Auto	Host	-11.1153
1	MC	NB	126	Con. Auto	Host	-15.627
1	MC	NB	130	Con. Auto	Host	-13.2507
1	MC	NB	138	Con. Auto	Host	-17.3043
1	MC	NB	140	Con. Auto	Host	-17.2871
1	MC	NB	126	Con. Hetero	Host	-16.2508
1	MC	NB	130	Con. Hetero	Host	-16.0946
1	MC	NB	138	Con. Hetero	Host	-17.4231
1	MC	NB	140	Con. Hetero	Host	-15.3393
1	MC	NB	126	Hetero	Host	-16.5294
1	MC	NB	130	Hetero	Host	-15.1371
1	MC	NB	138	Hetero	Host	-17.5994
1	MC	NB	140	Hetero	Host	-16.369
1	MC	B	127	Auto	Symb	-5.13277
1	MC	B	131	Auto	Symb	-0.26933
1	MC	B	139	Auto	Symb	-3.73647
1	MC	B	127	Con. Auto	Symb	-13.563
1	MC	B	131	Con. Auto	Symb	-12.091
1	MC	B	139	Con. Auto	Symb	-14.3021
1	MC	B	141	Con. Auto	Symb	-14.3169

1	MC	B	127	Con. Hetero	Symb	-13.5626
1	MC	B	131	Con. Hetero	Symb	-16.2815
1	MC	B	141	Con. Hetero	Symb	-14.4904
1	MC	B	127	Hetero	Symb	-12.377
1	MC	B	131	Hetero	Symb	-13.3119
1	MC	B	139	Hetero	Symb	-13.4319
1	MC	B	141	Hetero	Symb	-15.3821
1	MC	NB	126	Auto	Symb	-4.99355
1	MC	NB	130	Auto	Symb	3.290523
1	MC	NB	138	Auto	Symb	-17.231
1	MC	NB	126	Con. Auto	Symb	-15.5215
1	MC	NB	130	Con. Auto	Symb	-13.3076
1	MC	NB	138	Con. Auto	Symb	-16.1493
1	MC	NB	140	Con. Auto	Symb	-15.5845
1	MC	NB	126	Con. Hetero	Symb	-15.7415
1	MC	NB	130	Con. Hetero	Symb	-16.1941
1	MC	NB	138	Con. Hetero	Symb	-17.1641
1	MC	NB	140	Con. Hetero	Symb	-14.624
1	MC	NB	126	Hetero	Symb	-16.0543
1	MC	NB	138	Hetero	Symb	-16.7781
1	MC	NB	140	Hetero	Symb	-16.3871
4-5	MC	B	127	Auto	Bundles	-9.81155
4-5	MC	B	127	Con. Auto	Bundles	-14.4806
4-5	MC	NB	126	Auto	Bundles	-10.1526
4-5	MC	NB	138	Auto	Bundles	-12.9037
4-5	MC	NB	126	Con. Auto	Bundles	-17.0678
4-5	MC	NB	138	Con. Auto	Bundles	-17.4589
3-4	MC	NB	126	Con. Hetero	Bundles	-17.1816
3-4	MC	NB	138	Con. Hetero	Bundles	-17.168
3-4	MC	NB	126	Hetero	Bundles	-17.1738
3-4	MC	NB	138	Hetero	Bundles	-16.8644
4-5	MC	NB	126	Auto	Eggs	-12.3977
4-5	MC	NB	138	Auto	Eggs	-13.3323
4-5	MC	NB	126	Con. Auto	Eggs	-16.6604
4-5	MC	NB	138	Con. Auto	Eggs	-17.5205
4-5	MC	NB	140	Con. Auto	Eggs	-15.4996
3-4	MC	NB	126	Con. Hetero	Eggs	-16.2112
3-4	MC	NB	140	Con. Hetero	Eggs	-16.2192
3-4	MC	NB	126	Hetero	Eggs	-17.0758
3-4	MC	NB	138	Hetero	Eggs	-16.3921
3-4	MC	NB	140	Hetero	Eggs	-16.4337
7	MC	B	127	Auto	Host	-9.3514
7	MC	B	139	Auto	Host	-12.5603
7	MC	B	141	Auto	Host	-13.2668

7	MC	B	127	Con. Auto	Host	-9.03872
7	MC	B	131	Con. Auto	Host	-8.17055
7	MC	B	139	Con. Auto	Host	-11.8349
7	MC	B	141	Con. Auto	Host	-8.48092
7	MC	B	127	Con. Hetero	Host	-16.4261
7	MC	B	139	Con. Hetero	Host	-15.1433
7	MC	B	127	Hetero	Host	-12.5696
7	MC	B	131	Hetero	Host	-13.9792
7	MC	B	139	Hetero	Host	-14.6693
7	MC	B	141	Hetero	Host	-16.3557
7	MC	NB	126	Auto	Host	-10.5541
7	MC	NB	130	Auto	Host	-11.0545
7	MC	NB	138	Auto	Host	-14.7472
7	MC	NB	140	Auto	Host	-10.344
7	MC	NB	130	Con. Auto	Host	-14.7215
7	MC	NB	138	Con. Auto	Host	-16.896
7	MC	NB	140	Con. Auto	Host	-16.2512
7	MC	NB	126	Con. Auto	Host	-16.2829
7	MC	NB	138	Con. Hetero	Host	-9.23368
7	MC	NB	130	Con. Hetero	Host	-14.2367
7	MC	NB	140	Hetero	Host	-16.0552
7	MC	NB	126	Hetero	Host	-14.2335
7	MC	NB	138	Hetero	Host	-15.8566
7	MC	B	127	Auto	Eggs	-9.77349
7	MC	B	131	Auto	Eggs	-7.90568
7	MC	B	139	Auto	Eggs	-11.8139
7	MC	B	141	Auto	Eggs	-7.93339
7	MC	B	127	Con. Auto	Eggs	-13.6992
7	MC	B	131	Con. Auto	Eggs	-14.003
7	MC	B	139	Con. Auto	Eggs	-15.2008
7	MC	B	141	Con. Auto	Eggs	-14.0992
7	MC	B	127	Con. Hetero	Eggs	-14.0574
7	MC	B	131	Con. Hetero	Eggs	-14.7234
7	MC	B	139	Con. Hetero	Eggs	-15.39
7	MC	B	141	Con. Hetero	Eggs	-14.3146
7	MC	B	127	Hetero	Eggs	-14.3425
7	MC	B	131	Hetero	Eggs	-15.1795
7	MC	B	139	Hetero	Eggs	-15.4493
7	MC	B	141	Hetero	Eggs	-16.669
7	MC	NB	126	Auto	Eggs	-14.4314
7	MC	NB	130	Auto	Eggs	-11.421
7	MC	NB	138	Auto	Eggs	-13.6879
7	MC	NB	140	Auto	Eggs	-7.97131
7	MC	NB	126	Con. Auto	Eggs	-16.8229

7	MC	NB	130	Con. Auto	Eggs	-14.688
7	MC	NB	138	Con. Auto	Eggs	-16.9896
7	MC	NB	140	Con. Auto	Eggs	-17.0754
7	MC	NB	126	Con. Hetero	Eggs	-19.8491
7	MC	NB	130	Con. Hetero	Eggs	-14.6705
7	MC	NB	138	Con. Hetero	Eggs	-16.3066
7	MC	NB	140	Con. Hetero	Eggs	-16.1975
7	MC	NB	126	Hetero	Eggs	-14.4038
7	MC	NB	130	Hetero	Eggs	-13.4011
7	MC	NB	138	Hetero	Eggs	-15.4347
7	MC	NB	140	Hetero	Eggs	-16.9952
7	MC	B	127	Auto	Symb	-6.32771
7	MC	B	139	Auto	Symb	-8.80472
7	MC	B	141	Auto	Symb	-13.6898
7	MC	B	127	Con. Auto	Symb	-7.89861
7	MC	B	131	Con. Auto	Symb	-6.90889
7	MC	B	139	Con. Auto	Symb	-9.75123
7	MC	B	141	Con. Auto	Symb	-8.02497
7	MC	B	127	Con. Hetero	Symb	-16.6904
7	MC	B	139	Con. Hetero	Symb	-15.4244
7	MC	B	127	Hetero	Symb	-12.324
7	MC	B	131	Hetero	Symb	-14.5904
7	MC	B	139	Hetero	Symb	-13.3329
7	MC	NB	126	Auto	Symb	-7.07731
7	MC	NB	130	Auto	Symb	-7.93914
7	MC	NB	138	Auto	Symb	-12.5465
7	MC	NB	140	Auto	Symb	-5.59531
7	MC	NB	130	Con. Auto	Symb	-14.487
7	MC	NB	138	Con. Auto	Symb	-17.0642
7	MC	NB	140	Con. Auto	Symb	-15.643
7	MC	NB	126	Con. Auto	Symb	-15.9395
7	MC	NB	130	Con. Hetero	Symb	-15.3648
7	MC	NB	138	Con. Hetero	Symb	-6.51092
7	MC	NB	138	Hetero	Symb	-13.7296
7	MC	NB	140	Hetero	Symb	-15.6207
7	MC	NB	126	Hetero	Symb	-17.0631
7	MC	NB	130	Hetero	Symb	-13.3824
1	PC	B	157	Auto	Host	-15.135
1	PC	B	161	Auto	Host	-12.0174
1	PC	B	159	Auto	Host	-9.41657
1	PC	B	169	Auto	Host	-10.8329
1	PC	B	159	Con. Auto	Host	-15.2819
1	PC	B	161	Con. Auto	Host	-15.5362
1	PC	B	169	Con. Auto	Host	-16.9658

1	PC	B	159	Con. Hetero	Host	-17.4982
1	PC	B	157	Con. Hetero	Host	-15.8828
1	PC	B	161	Con. Hetero	Host	-15.5599
1	PC	B	159	Hetero	Host	-16.5073
1	PC	B	161	Hetero	Host	-15.352
1	PC	B	157	Hetero	Host	-15.9387
1	PC	B	169	Hetero	Host	-15.7403
1	PC	NB	166	Auto	Host	-14.9127
1	PC	NB	160	Auto	Host	-11.4487
1	PC	NB	162	Auto	Host	-14.9893
1	PC	NB	158	Auto	Host	-8.93881
1	PC	NB	162	Con. Auto	Host	-15.5248
1	PC	NB	166	Con. Auto	Host	-16.4828
1	PC	NB	158	Con. Auto	Host	-12.4759
1	PC	NB	160	Con. Auto	Host	-16.3354
1	PC	NB	158	Con. Hetero	Host	-15.2203
1	PC	NB	170	Con. Hetero	Host	-16.0896
1	PC	NB	160	Con. Hetero	Host	-15.7537
1	PC	NB	162	Con. Hetero	Host	-15.4714
1	PC	NB	158	Hetero	Host	-14.5989
1	PC	NB	170	Hetero	Host	-16.0703
1	PC	NB	160	Hetero	Host	-15.1649
1	PC	NB	162	Hetero	Host	-15.6929
1	PC	B	157	Auto	Symb	-13.0451
1	PC	B	161	Auto	Symb	-7.54882
1	PC	B	159	Auto	Symb	0.714964
1	PC	B	169	Auto	Symb	-1.98219
1	PC	B	159	Con. Auto	Symb	-16.4413
1	PC	B	161	Con. Auto	Symb	-15.3114
1	PC	B	169	Con. Auto	Symb	-16.2906
1	PC	B	159	Con. Hetero	Symb	-16.3587
1	PC	B	157	Con. Hetero	Symb	-14.5615
1	PC	B	161	Con. Hetero	Symb	-15.1198
1	PC	B	159	Hetero	Symb	-16.0561
1	PC	B	161	Hetero	Symb	-14.9537
1	PC	B	157	Hetero	Symb	-15.3614
1	PC	B	169	Hetero	Symb	-14.4336
1	PC	NB	166	Auto	Symb	-13.8323
1	PC	NB	160	Auto	Symb	-3.89456
1	PC	NB	162	Auto	Symb	-14.9849
1	PC	NB	158	Auto	Symb	-0.64484
1	PC	NB	162	Con. Auto	Symb	-14.3583
1	PC	NB	166	Con. Auto	Symb	-15.6162
1	PC	NB	160	Con. Auto	Symb	-14.7027

1	PC	NB	158	Con. Hetero	Symb	-15.0209
1	PC	NB	170	Con. Hetero	Symb	-15.2673
1	PC	NB	160	Con. Hetero	Symb	-15.191
1	PC	NB	162	Con. Hetero	Symb	-14.6922
1	PC	NB	158	Hetero	Symb	-12.9995
1	PC	NB	170	Hetero	Symb	-15.4915
1	PC	NB	160	Hetero	Symb	-13.3143
1	PC	NB	162	Hetero	Symb	-12.7243
7	PC	B	157	Auto	Host	-12.9443
7	PC	B	169	Auto	Host	-10.0412
7	PC	B	159	Auto	Host	-11.4849
7	PC	B	169	Con. Auto	Host	-17.1296
7	PC	B	157	Con. Auto	Host	-11.5933
7	PC	B	159	Con. Auto	Host	-15.6601
7	PC	B	161	Con. Auto	Host	-13.3123
7	PC	B	161	Con. Hetero	Host	-15.6973
7	PC	B	157	Con. Hetero	Host	-15.3102
7	PC	B	169	Con. Hetero	Host	-16.8426
7	PC	B	159	Con. Hetero	Host	-17.7066
7	PC	B	161	Hetero	Host	-15.8001
7	PC	B	169	Hetero	Host	-16.8153
7	PC	B	159	Hetero	Host	-15.6494
7	PC	B	157	Hetero	Host	-15.2817
7	PC	NB	170	Auto	Host	-15.1145
7	PC	NB	158	Auto	Host	-11.4721
7	PC	NB	160	Auto	Host	-12.666
7	PC	NB	161	Auto	Host	-15.827
7	PC	NB	162	Con. Auto	Host	-15.0318
7	PC	NB	160	Con. Auto	Host	-15.4035
7	PC	NB	158	Con. Auto	Host	-14.8609
7	PC	NB	170	Con. Hetero	Host	-15.4093
7	PC	NB	160	Con. Hetero	Host	-16.1125
7	PC	NB	158	Hetero	Host	-14.7958
7	PC	NB	160	Hetero	Host	-16.119
7	PC	NB	162	Hetero	Host	-15.3669
7	PC	NB	170	Hetero	Host	-16.2676
7	PC	B	169	Auto	Eggs	-12.13
7	PC	B	161	Auto	Eggs	-13.1788
7	PC	B	159	Con. Auto	Eggs	-15.2414
7	PC	B	161	Con. Auto	Eggs	-14.8263
7	PC	B	169	Con. Auto	Eggs	-17.1616
7	PC	B	161	Con. Hetero	Eggs	-15.8689
7	PC	B	169	Con. Hetero	Eggs	-15.3517
7	PC	B	159	Hetero	Eggs	-16.9584

7	PC	B	161	Hetero	Eggs	-16.9866
7	PC	B	169	Hetero	Eggs	-16.8247
7	PC	NB	158	Auto	Eggs	-6.90
7	PC	NB	160	Auto	Eggs	-2.17
7	PC	NB	162	Auto	Eggs	-3.35
7	PC	NB	170	Auto	Eggs	-5.73
7	PC	NB	158	Con. Auto	Eggs	-10.16
7	PC	NB	160	Con. Auto	Eggs	-14.60
7	PC	NB	162	Con. Auto	Eggs	-19.43
7	PC	NB	170	Con. Auto	Eggs	-16.20
7	PC	NB	158	Con. Hetero	Eggs	-10.03
7	PC	NB	160	Con. Hetero	Eggs	-12.21
7	PC	NB	162	Con. Hetero	Eggs	-11.15
7	PC	NB	170	Con. Hetero	Eggs	-11.15
7	PC	NB	158	Hetero	Eggs	0.10
7	PC	NB	160	Hetero	Eggs	-4.96
7	PC	NB	162	Hetero	Eggs	-13.89
7	PC	NB	170	Hetero	Eggs	-10.92
7	PC	B	157	Auto	Symb	-9.78596
7	PC	B	169	Auto	Symb	-9.99739
7	PC	B	159	Auto	Symb	-7.71707
7	PC	B	169	Con. Auto	Symb	-16.6776
7	PC	B	159	Con. Auto	Symb	-14.7313
7	PC	B	161	Con. Auto	Symb	-12.338
7	PC	B	161	Con. Hetero	Symb	-14.2532
7	PC	B	157	Con. Hetero	Symb	-15.4105
7	PC	B	169	Con. Hetero	Symb	-15.5703
7	PC	B	159	Con. Hetero	Symb	-16.493
7	PC	B	161	Hetero	Symb	-15.1101
7	PC	B	169	Hetero	Symb	-16.3476
7	PC	B	159	Hetero	Symb	-14.9936
7	PC	B	157	Hetero	Symb	-14.0338
7	PC	NB	170	Auto	Symb	-12.372
7	PC	NB	158	Auto	Symb	-7.57338
7	PC	NB	160	Auto	Symb	-8.81346
7	PC	NB	161	Auto	Symb	-15.8273
7	PC	NB	162	Con. Auto	Symb	-14.4793
7	PC	NB	160	Con. Auto	Symb	-14.5474
7	PC	NB	158	Con. Auto	Symb	-14.435
7	PC	NB	170	Con. Hetero	Symb	-15.1516
7	PC	NB	160	Con. Hetero	Symb	-13.4029
7	PC	NB	158	Hetero	Symb	-15.5522
7	PC	NB	160	Hetero	Symb	-16.259
7	PC	NB	162	Hetero	Symb	-15.6479

7	PC	NB	170	Hetero	Symb	-15.8874
